# Coroner’s Inquests

**Published:** 1870-03

**Authors:** 


### *Coroner's Inquests.*

IT is extremely to be regretted that, for unexplained reasons, the *post-mortem* examinations, made by order of the coroner, however they may conduce to the maintenance of justice and public order, are totally unprofitable toward scientific progress.

A case in point has recently occurred in this city. An unfortunate colored man received a wound in the axillary region, the hæmorrhage from which was so profuse as to require surgical interference. A thoroughly experienced and highly accomplished surgeon proposed to enlarge the opening and tie the artery at the point. The colored man, declining this operation, soon after fell into the hands of some parties, to us unknown, who proposed some treatment (also unknown), which, it seems, was unsuccessful, as, subsequently, an attempt was made to ligate the subclavian. Hence, a speedy coroner's inquest, with a verdict of *death from hæmorrhage.*

What was found by the official examiner was, briefly:

"Inferior trunks of axillary plexus torn up. Could not find the phrenic nerve.

"Ligature included full half of the scalenus anticus muscle, as well as the artery.

"On opening the thorax, the lung was found collapsed, and a broad beam of daylight poured into the cavity from the seat of the operation in the neck."

Under these circumstances, the verdict given strikes us as requiring some explanation. *Quien sabe?*

